# A Miniature Fibre-Optic Raman Probe Fabricated by Ultrafast Laser-Assisted Etching

**DOI:** 10.3390/mi11020185

**Published:** 2020-02-11

**Authors:** Calum A. Ross, David G. MacLachlan, Brian J. E. Smith, Rainer J. Beck, Jonathan D. Shephard, Nick Weston, Robert R. Thomson

**Affiliations:** 1 Scottish Universities Physics Alliance (SUPA), Institute of Photonics and Quantum Sciences (IPaQS), Heriot-Watt University, Edinburgh EH14 4AS, UK; 2 Renishaw Plc, New Mills, Wotton-under-Edge GL12 8JR, UK; 3 Renishaw Plc, Edinburgh EH14 4AP, UK; 4 EPSRC IRC Hub, MRC Centre for Inflammation Research, Queen’s Medical Research Institute (QMRI), University of Edinburgh, Edinburgh EH16 4TJ, UK

**Keywords:** optical biopsy, Raman spectroscopy, micro-optics, ultrafast laser-assisted etching

## Abstract

Optical biopsy describes a range of medical procedures in which light is used to investigate disease in the body, often in hard-to-reach regions via optical fibres. Optical biopsies can reveal a multitude of diagnostic information to aid therapeutic diagnosis and treatment with higher specificity and shorter delay than traditional surgical techniques. One specific type of optical biopsy relies on Raman spectroscopy to differentiate tissue types at the molecular level and has been used successfully to stage cancer. However, complex micro-optical systems are usually needed at the distal end to optimise the signal-to-noise properties of the Raman signal collected. Manufacturing these devices, particularly in a way suitable for large scale adoption, remains a critical challenge. In this paper, we describe a novel fibre-fed micro-optic system designed for efficient signal delivery and collection during a Raman spectroscopy-based optical biopsy. Crucially, we fabricate the device using a direct-laser-writing technique known as ultrafast laser-assisted etching which is scalable and allows components to be aligned passively. The Raman probe has a sub-millimetre diameter and offers confocal signal collection with 71.3% ± 1.5% collection efficiency over a 0.8 numerical aperture. Proof of concept spectral measurements were performed on mouse intestinal tissue and compared with results obtained using a commercial Raman microscope.

## 1. Introduction

Delayed diagnosis, often due to the late onset of recognisable symptoms, is a major contributor to the high mortality rates of many cancers. In particular, cancers that develop deep within the body such as oesophageal, bowel, and lung cancer may not present noticeable symptoms until the cancer is already at an aggressive stage. Oesophageal cancer (OC) has a particularly poor prognosis, with a five-year survival rate of between 12% and 18% [1]. OC is commonly diagnosed by a surgical endoscopic biopsy followed by histopathology; however, clinicians must rely on very subtle guidance from white-light endomicroscopy and, therefore, biopsy sampling can be poor. Further, histopathology is complex and time-consuming, and staging the cancer can be subjective. To improve the overall prognosis of OC and other cancers, a diagnostic tool that is minimally invasive and provides real-time tissue classification at an early stage of cancer development is needed. 

Over the past two decades, optical biopsy techniques [2] have progressed from a research environment into the clinic, and clinical studies in which optical techniques are used to aid decision making at point-of-care are now underway. The term “optical biopsy” describes several techniques that use light to determine clinically relevant tissue information and includes endomicroscopy, optical coherence tomography (OCT), fluorescence endoscopy, and spectroscopy amongst others—often performed in hard-to-reach regions of the body via optical fibres. Fibre-based Raman spectroscopy is a specific example, which has been successful in clinical studies for differentiating normal and cancerous tissue in vivo in several regions of the body including the oesophagus, lungs, skin, and brain [3,4,5,6,7,8]. A Raman-based optical biopsy relies on the collection of photons scattered inelastically by molecules present in tissue. Every molecule has several associated vibrational modes, depending on the number of atoms present, and each mode has a discreet vibrational energy. An inelastically scattered photon exchanges this energy with the vibrational mode, resulting in a spectral shift known as a “Raman shift”. Collectively, a group of photons scattered inelastically by tissue carry a spectral “fingerprint” that infers the molecular makeup of the tissue. Since tissue is molecularly complex, very subtle variations between tissues can be detected by performing multivariate analysis on the Raman-shifted spectra, and, therefore, normal tissue can be distinguished from precancer and cancer tissue [9,10,11]. 

To perform Raman spectroscopy deep inside the body, optical fibres are used to transport light to and from the target site. Often, miniaturised optics are needed at the distal end to direct the excitation light onto the tissue and collect Raman-scattered light back into return fibres efficiently. Many distal-end optical system (DOS) configurations exist, but they typically consist of microlenses, filters, mirrors, and alignment features; all packaged into medical grade housing suitable for endoscopy. Although several groups have developed Raman probes [12,13,14], the fabrication and assembly of the microcomponents remain challenging, often requiring labour intensive and intricate manual alignment, which is ultimately unsuitable for large-scale manufacture. Additionally, the complexity of the optical system may be limited by conventional optics, which is a particular hindrance for probes with an outer diameter of less than 1 mm. 

Addressing the manufacturing limitations, we propose that a direct-laser-writing technique called ultrafast laser-assisted etching (ULAE) is ideally suited towards the fabrication of DOS’s for Raman probe instruments, overcoming many of the shortfalls of traditional techniques. ULAE is a two-step process in which components are first inscribed in a bulk fused silica chip using focused femtosecond laser pulses. When optimal irradiation parameters are used, the laser-induced modification enhances the local etchability of the silica by up to three orders of magnitude [15,16], allowing the laser-inscribed material to be subsequently removed in a second, chemical etching step. Within the last two decades, collaborative efforts towards better understanding the light–matter interactions [17,18,19] and chemical etching mechanisms [20] and advanced writing techniques [21,22] have established ULAE as a capable manufacturing method for complex glass microcomponents and systems [23,24]. Recently, ULAE has shifted away from using the widely adopted but notoriously hazardous etching agent hydrofluoric acid (HF) to potassium hydroxide (KOH), making the technique much more appealing for industry-level manufacturing. ULAE is particularly suited to fabricating systems of micro-optics because multiple components can be written onto a single chip in three dimensions with an alignment tolerance set by the precision of the stages that translate the material through the laser focus—typically tens of nanometres for modern stages. 

In this paper, we describe the development and fabrication of a miniaturised Raman probe with a novel DOS design fabricated by ULAE. First, we give a brief overview of some existing Raman probes and discuss the design considerations that led to the development of the system presented here. Optical modelling is provided along with specifics of the fabrication process including a flame polishing technique developed to improve the surface quality of ULAE fabricated lenses. Finally, the performance of the probe is investigated, including proof-of-concept Raman analysis of cancerous mouse intestine tissue. 

## 2. Raman Probe State of the Art 

This brief review will summarise some of the fibre-based Raman probes that have been developed recently for optical biopsy applications. Raman probe design has been reviewed in detail by other groups [25,26], and a thorough review of all aspects of probe development, including clinical integration, is given by Stevens et al. [27]. The function of a Raman probe is to deliver monochromatic excitation (or pump) light to the intended tissue site and collect Raman-scattered light, while at the same time rejecting elastically scattered and reflected pump light from returning. Raman probes vary in complexity from a simple single optical fibre without distal-end optics to intricate systems of several fibres to separate the pump light and Raman signal and multi-element distal-end optical systems to improve signal collection efficiency. Generally, the complexity of a Raman probe is a trade-off between manufacturability and performance. 

Single-fibre Raman probes without distal-end optics are inexpensive, easy to manufacture, and have a small outer diameter. However, the fibre material itself, commonly silica, produces a Raman signal as the pump light propagates along it (referred to as the fibre background, FB) which can be several orders of magnitude greater than the tissue Raman signal. Recent advances in fibre manufacturing have opened up new avenues to address this; notably, non-standard fibre materials such as sapphire [28] and microstructured, hollow-core fibres [29,30] have been shown to dramatically reduce the fibre background signal. Single-fibre probes still exhibit limited collection efficiency and so instead multicore fibres or fibre bundles are sometimes used to improve signal collection without increasing the complexity of manufacture significantly [31,32]. The arrangement of fibre bundles and a comparison of flat versus bevelled fibre tips was reviewed by Skinner et al. The review also describes the use of ball lenses to add directionality to the pump light at the distal end [33,34]. Ball lenses are commonly used to improve the collection efficiency of Raman probes [3,12] because they are relatively simple to align and assemble.

More complex distal-end optics can be added to improve probe performance in terms of collection efficiency, sampling range, and Raman signal isolation. Day et al. developed a confocal Raman probe capable of rejecting light originating beyond a confined sampling region [35], and later adapted the probe to fit within the bore of a hypodermic needle [13]. Probes for advanced Raman techniques such as spatially offset Raman spectroscopy (SORS) [36,37] and surface-enhanced Raman spectroscopy (SERS) [38] have also been developed. Many probes rely on forward axial sampling, whereas others include mirrors to deflect the signal laterally for side-viewing [39]. 

## 3. Probe Design

### 3.1. Design Considerations

The Raman probe described in this section was designed to take full advantage of the three-dimensional fabrication capabilities of ULAE. The first factor considered was the overall form factor of the distal probe; gastroscopes have a standard instrument channel diameter of 2.8 mm [40], however, for applications requiring an embedded needle probe, the optical system must be sub-millimetre. With traditional fabrication techniques, sub-mm probes are limited to very simple optics due to fabrication challenges, however, much more complex surfaces can be fabricated on this scale using ULAE. 

As mentioned, hollow-core fibres exhibit significantly reduced fibre background signals than solid-core fibres, but such fibres are still in relative infancy and are expensive as a result. Low-cost instruments are desirable in a clinical setting as single use is preferred. Therefore, solid-core fibres are more suitable currently. For our probe, the fibre background was instead minimised by using separate light-delivery and collections paths. 

The number and arrangement of return fibres is also an important consideration. Several collection cores surrounding a central pump fibre ensures symmetrical collection about the sampling volume and the collected light can be reformatted into a slit at the proximal end for efficient coupling to a spectrometer. We determined that a six-around-one configuration for light delivery and collection provided the highest collection efficiency. When paired with collimating and focusing lenses, each return fibre acts like a pinhole in a confocal microscopy system, only collecting light from a well-defined sampling volume shared with each other collection fibre. The ideal aperture size of the return cores is a trade-off between confocality and signal strength: Smaller cores increase the spatial sampling resolution of the probe whereas larger cores relax alignment tolerances.

Raman spectroscopy has the distinct advantage over several other spectroscopic techniques in that the positions of the spectral peaks in the Raman fingerprint are determined by the molecules present in the sample and are independent of the excitation wavelength. Therefore, Raman spectra collected with different systems and excitation sources can be easily compared and classified. With that said, there are considerations when choosing the optimum excitation wavelength. The probability of a Raman scattering event occurring is inversely proportional to the wavelength of the incident photons to the fourth power, suggesting that shorter wavelengths are a better choice for excitation. However, at shorter wavelengths, fluorescence can dominate the emission spectra, particularly for organic materials. Additionally, shorter wavelengths are absorbed by tissue more readily than longer wavelengths and so the maximum permitted pump power is reduced. Light of 785 nm wavelength offers a suitable trade-off between signal strength, fluorescence and absorbance and has become widely adopted for Raman spectroscopy, particularly for tissue analysis. As a result, compatible components are readily available and often cost effective. 

### 3.2. DOS Description and Modelling 

**Figure 1:**
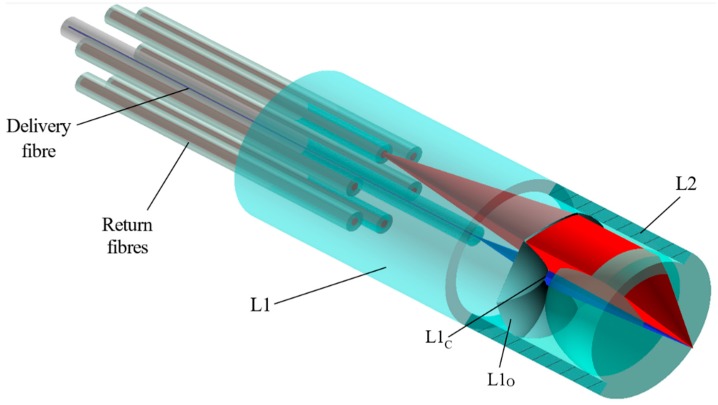
A partial cut-away rendering of the proposed distal-end optical system (DOS) for the laser-written Raman probe. The optical system consists of two components, L1 and L2, which interface with one light delivery and six light return optical fibres. Excitation light (represented in blue) is delivered by a central fibre and focused beyond the tip of the probe. Raman-scattered light (one sixth shown only—represented in red) is collected by the DOS and coupled back into the six return fibres for analysis.

A schematic of the proposed DOS is shown in Figure 1. The optical system consists of two components: a six-around-one arrangement of optical fibre alignment holes paired with a freeform “petal”-shaped lens array, L1, and a 0.8 numerical aperture (NA) plano-convex aspheric lens embedded in a surrounding cylindrical alignment sleeve, L2. The petal lens array consists of a central lens, L1_C_, surrounded by six equally spaced overlapping lenses, L1_O_. 

The optical system operates as follows: 785 nm pump light is delivered centrally via a 0.1 NA single-mode silica fibre and is collimated by L1_C_ and then focused by L2 into the specimen. Scattered light generated within the focal volume is then collected by L2 and directed back towards L1, which couples the light into six multimode collection fibres. L1 was designed such that the central lens used to collimate the pump light occupied only 4.2% of the petal lens surface area, leaving the remaining surface for Raman signal collection. In this configuration, a theoretical 85.1% of light scattered within the 0.8 NA of L2 is collected by the return fibres with loses primarily due to Fresnel reflections at the optical interfaces as determined by ray-trace simulations. The probe was designed to be used in contact with tissue with light collected from approximately 300 μm below the surface, coinciding with the region in which pre-cancers are expected to form in the epidermis [41]. L2 was designed with a low F-number so that the collection NA could remain large while keeping the overall probe diameter below 1 mm. This lens configuration also ensures a reasonable degree of confocality—light originating outside the intended sampling plane is rejected by the return optical fibres. This allows individual features in a complex inhomogeneous sample such as tissue to be probed independently.

**Figure 2:**
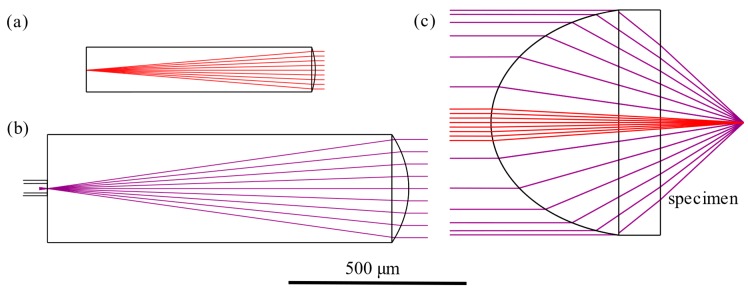
Ray diagrams for each of the three lens surfaces in the DOS: (**a**) L1_C_, (**b**) L1_O_, (**c**) L2. Each lens takes the form of a prolate asphere, which efficiently collimates or focuses the rays over the desired spectral range.

Each of the components was designed and optimised using Zemax Optic Studio ray tracing software (version 16.5, Zemax, Kirkland, WA, USA) to ensure that the signal transmission was maximised and the etendue of the system was preserved. Ray-trace diagrams for each of the three lens types, L1_C_, L1_O,_ and L2, are shown in Figure 2. Aspheric lenses were chosen in each case for optimum performance, with radii of 0.194, 0.411, and 0.291 mm and conic constants equal to −0.473, −0.473, and −0.490, respectively.

## 4. Probe Fabrication and Characterisation

### 4.1. Ultrafast Laser-Assisted Etching

Ultrafast laser-assisted etching (ULAE) is a three-dimensional micromanufacturing technique that relies on ultrashort laser pulses to modify a transparent material’s physical and chemical properties locally within a well-confined focal volume. Under certain laser irradiation conditions, the material can be modified such that its chemical etchability is significantly enhanced. Therefore, the laser-written material can be selectively removed in a subsequent etching step, facilitating the precise fabrication of almost arbitrary 3D structures on a scale spanning tens of microns to tens of millimetres. The technique can be applied to several transparent materials including fused silica, which is widely adopted in optics for its excellent optical and mechanical properties. Recently, etching rate enhancements of up to three orders of magnitude have been demonstrated in fused silica [16,42], paving the way for a new era in glass microfabrication. 

The microcomponents described here were fabricated in 1 mm thick high-purity fused silica wafers (UV Grade Corning 7980 0F, Corning Inc., Corning, NY, USA). Laser inscription was performed with a Menlo Systems Bluecut fibre laser (Menlo Systems GmbH, Martinsried, Germany), delivering 350 fs pulses at a repetition rate of 250 kHz and a central wavelength of 1030 nm. The laser pulse energy was set to approximately 200 nJ for the majority of the inscription and the polarisation was linear and orientated orthogonally to the intended etching direction in order to achieve the highest etching selectivity as discussed in [16]. The substrate was translated on a motorised three-axis crossed roller bearing stage (Alio Industries, Arvada, CO, USA) with up to 5 nm positioning resolution. The stages were controlled via an EtherCAT controller (ACS motion control, Migdal Ha-Emek, Israel), and the stage motion was programmed in the proprietary computer numerical control (CNC) programming language ASCPL+.

In the subsequent etching step, the substrates were submerged in an etching solution of KOH diluted with deionised water to a concentration of 8 mol/L and heated to 85° C. The etchant was magnetically stirred for the duration of the etch to ensure the solution remained homogeneous. Afterwards, the components were rinsed thoroughly in deionised water before inspection. 

**Figure 3:**
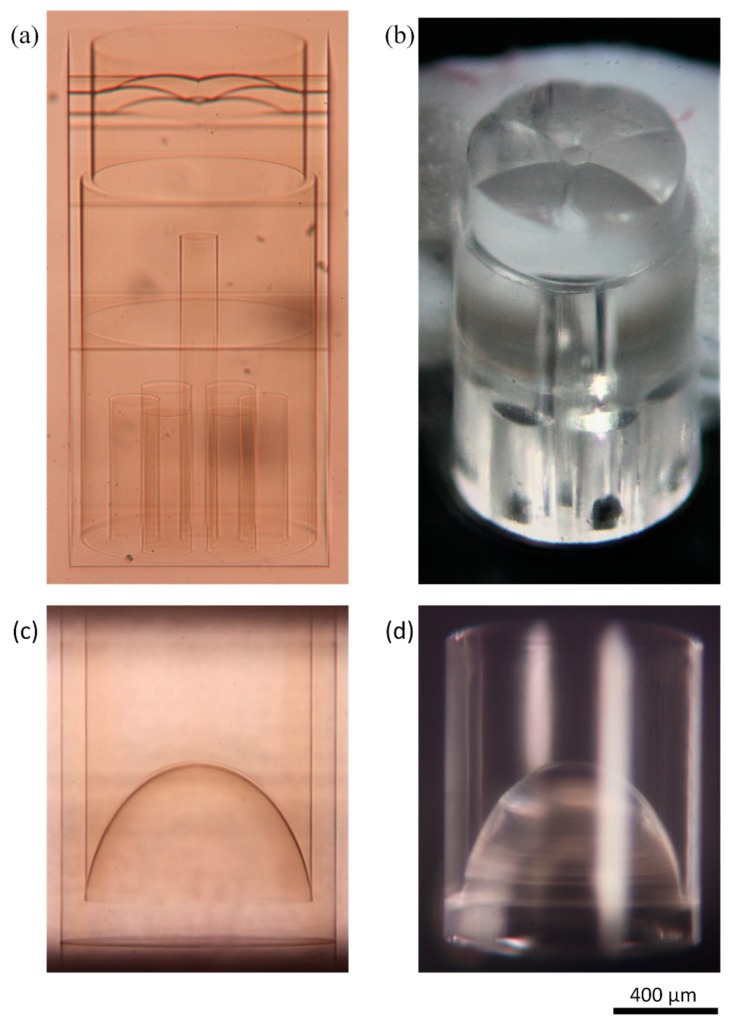
Micrographs of the two distal-end probe components L1 (**a**,**b**) and L2 (**c**,**d**) after ultrafast laser inscription (**a**,**c**) and after etching (**b**,**d**). The outer diameter of each component was 960 μm.

The components were inscribed by raster scanning the laser focus to form the individual component surfaces. Additional etch planes and channels were written into the substrate, as shown in Figure 3a, to minimise the etching time and allow the parts to separate in the etching bath. An alternative approach is occasionally described in literature, whereby the material to be removed is laser-written volumetrically and then etched away uniformly [42]. Advantages of this method are that the CNC code is typically simpler and the laser polarisation can remain fixed; however, disadvantages include longer inscription times and a rougher surface finish, which is not suitable for lens manufacture. Micrographs of the two probe components taken both after laser inscription and after etching are presented in Figure 3.

### 4.2. Flame Polishing

After fabrication, etched surfaces exhibit roughness and nano-texturing carried through from the laser inscription stage. The roughness is typically on the order of hundreds of nanometres, which is acceptable for alignment features, but introduces detrimental scattering for optical surfaces. The scattering has a minimal effect on the overall throughput of the lenses but can lead to unwanted background signals entering the return fibres and may introduce noise in the Raman spectra data. To reduce the surface roughness, we developed a flame polishing technique in which the lens surface was directly exposed to a controlled microflame for a short time period in order to melt and re-flow a thin layer on the surface. Flame polishing was performed using an AQUAFLAME SYSTEMS MODEL 500 (Aquaflame Systems, Birmingham, UK) oxyhydrogen torch with a 3 mm microflame generated through a 21-gauge nozzle. The generator produced hydrogen fuel via the electrolysis of deionised water and potassium hydroxide and the gas was passed through a solution of methyl ethyl ketone (MEK) to generate a flame with a peak temperature of 1850 °C, sufficiently beyond the softening point of fused silica to induce surface melting. When properly controlled, only the high spatial frequency surface deviations associated with roughness melted during polishing, and so the nominal form of the lens remained unaffected. The optimum polishing time was found experimentally by polishing several identical lenses for a varying amount of time. We found that the optimum time for the six-around-one lens was 0.25 s; however we also noted that the optimum time varied significantly depending on the component’s size and shape. 

**Figure 4:**
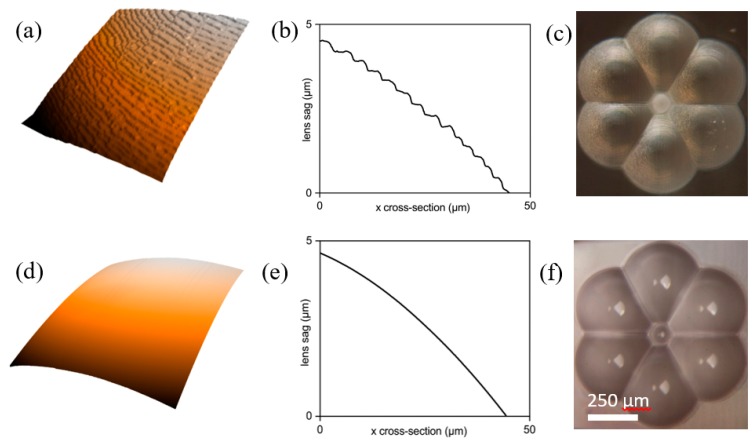
Example lens profiles measured before and after flame polishing using an atomic force microscope (AFM). (**a**–**c**) Shows a 3D colour plot, 2D surface profile, and micrograph, respectively before flame polishing. (**d**–**f**) Shows the same plots made after flame polishing for 0.25 s. The colour maps in (**a**,**d**) were measured over 50 μm × 50 μm areas. The arithmetic roughness, *R*_a_, was reduced from 48.7 to 2.26 nm.

An atomic force microscope (AFM) was used to measure the roughness of lenses before and after flame polishing and the results are presented in Figure 4. 

Figure 4a–c shows the surface profile of a lens before any polishing or post processing. The colourmap (Figure 4a) highlights the surface texturing caused by the imprint of nanogratings formed during laser writing [43]. After flame polishing, the nanotexturing was no longer apparent and the surface was significantly smoother as shown in Figure 4d–f. To quantify the reduction in surface roughness, the arithmetic average deviation of the surface from the nominal form, or mean roughness, *R*_a_, was measured. Before flame polishing, the lens was found to have a mean roughness of 48.7 nm, which was reduced to 2.26 nm after flame polishing for 0.25 s. Flame polishing was found to consistently reduce mean surface roughness down to a few nanometres, and, therefore, scattering is expected to be inconsequential over the operating wavelength (785–950 nm).

### 4.3. Probe Assembly and Spectrometer Coupling

**Figure 5:**
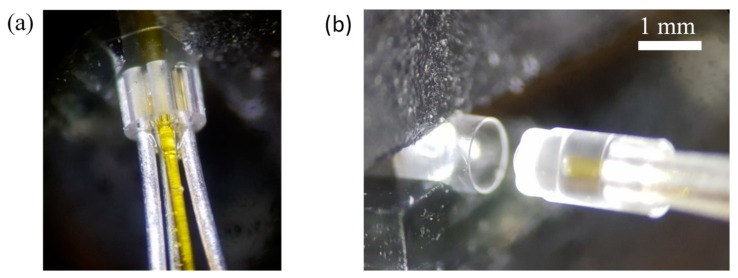
(**a**) The single delivery fibre and six return fibres were manually inserted into the passive alignment slots using precision translation stages and fixed in place with a UV-cured optical adhesive. (**b**) Similarly, the two distal-end probe components were joined and UV cured, with optical alignment achieved passively.

The distal-end optics and delivery and return fibres were assembled using manual precision translation stages and bonded using ultraviolet (UV) cured optical adhesive (Norland, NOA 61, Norland Products, Inc., Cranbury, NJ, USA). The alignment of each of the components was achieved passively using alignment slots laser written and etched directly into the optics. Micrographs showing the distal-end assembly process are presented in Figure 5. Each fibre was inserted and glued sequentially, with glue deposited onto the end of the fibre before inserting into a fibre alignment slot and UV cured. As well as bonding the fibres in position, the adhesive acted as a refractive index matching medium between the fibre tip and the end of the slot to reduce Fresnel reflection and scattering. The two distal-end optical components were bonded using a similar method with glue applied to the termination rim of L1, which was inserted into L2 before curing.

The Raman probe was packaged in either flexible heat-shrink tubing or within the bore of a 17-gauge blunt hypodermic needle for protection during experiments. 

**Figure 6:**
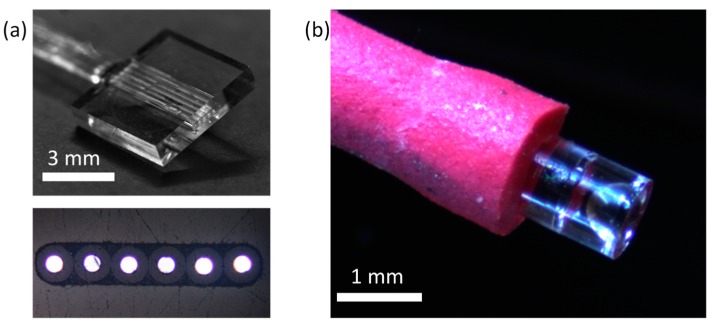
(**a**) At the proximal end, the return fibres (shown here with 50 μm diameter cores), were re-arranged into a linear array using an ultrafast laser-assisted etching (ULAE)-fabricated precision slot for efficient interfacing with traditional spectrometers. (**b**) A micrograph of the assembled distal end with heat-shrink tubing added for protection.

Collecting the Raman signal via six return fibres improves collection efficiency but poses a challenge of how to interface the fibres with a spectrometer efficiently. For optimal coupling, the six return fibres must be arranged into a linear array, which can be imaged onto the input slit of a spectrometer. To suitably arrange the return fibres, we developed a fibre reformatter with a precision slot into which the six fibres were fed through and bonded in position. The 5 mm long slot had a rounded rectangular aperture that confined the six return fibres laterally and angularly. The reformatter, presented in Figure 6a, was fabricated out of fused silica using the same laser-induced selective etching method as used for the distal optics to obtain the high-dimensional tolerance required to align the fibres precisely. The fibres, left unstripped for robustness, were fed through the slot and fixed in place with a UV-cured adhesive and ground and polished flat. The slot aligned the six fibres in a line within a lateral tolerance of ±2 μm. 

### 4.4. Fibre Alignment

Passive alignment of the optical components is critical for the manufacture of the Raman probe to be scalable and automated. The passive alignment slots were designed to accept stripped optical fibres within their manufacturing tolerance, typically ±1 μm. Any significant off-axis misalignment of the return fibres would result in a decrease in the optical fibre coupling of the return Raman signal. An angular misalignment is less detrimental to performance because the coupling lenses were designed with a slightly larger NA than the return fibres.

**Figure 7:**
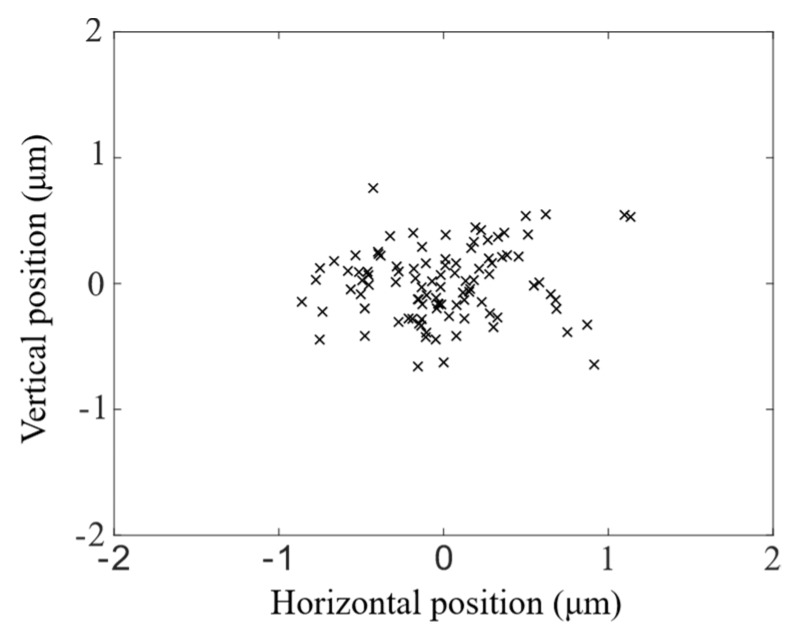
A scatter plot mapping the lateral positions of an optical fibre randomly inserted into an alignment slot 100 times. The alignment slot confined the fibre to within ±0.83 and ±0.52 μm of the optical axis in the horizontal and vertical axes respectively to two standard deviations.

To quantify the alignment precision of the laser-written fibre slots, a single-mode (SM) optical fibre was manually inserted one-hundred times into an alignment slot and its output imaged on a camera. The magnification of the imaging system was calibrated by imaging a multicore fibre with a measured core separation. The position of the SM fibre core was measured after each insertion and plotted on a scatter plot, shown in Figure 7. The lateral fibre position was confined to within ±0.83 and ±0.52 μm in the horizontal and vertical axes respectively to two standard deviations, or 95.45% of fibre insertions. The larger horizontal variation was due to an ellipticity of the alignment slot caused by the fabrication process but could be reduced by refining the process. The deviations were small in comparison to the 50 μm return fibre cores, and indeed ray-trace simulations showed that a lateral fibre misalignment of 1 μm resulted in no perceptible decrease in signal collection.

### 4.5. Collection Efficiency

The Raman probe was designed to collect light from a confined on-axis region approximately 300 μm beyond the probe tip over a 0.8 NA. The collection efficiency of the optical system is proportional to the signal-to-noise ratio (SNR) of the measured Raman signal, and a high efficiency is needed for the probe to operate within acceptable optical power limits and signal acquisition times. Here, we define the probe’s collection efficiency as the percentage of light originating from a point source over the probe NA which is collected by the six return fibres. In theory, the only losses in the system are due to Fresnel reflections at each optical interface and the areal portion of the petal lens that is used for light delivery and not collection. Optical simulations were performed to predict the theoretical throughput of the distal-end optical system and, after considering losses at the three optical interfaces and the return fibre coupling efficiency, the throughput was 85.1%. 

In practice, imperfect surfaces, minor misalignments, and “dead space” between lenses in the petal array also contribute to loss of signal. To measure the collection efficiency experimentally, a 785 nm “point source” was generated by passing a collimated beam through a variable NA immersion objective and collected by the probe. Water was used for immersion to approximate the refractive index of tissue and the probe was positioned to maximise the light collected by the six return fibres simultaneously. The NA of the objective was increased by opening an input aperture until the signal collected plateaued. The experimental efficiency was determined by comparing the collected light to the total power transmitted through the objective as measured with a photodiode and found to be 71.3% ± 1.5%. Slightly less than the theoretical maximum, the loss in collected light was attributed to scattering from the lens surfaces and minor misalignments of the optical components, which is expected within a tolerance set by the fabrication. 

## 5. Demonstration of Raman Spectra Acquisition of Tissue

To test the performance of the probe for Raman spectroscopy, the probe was integrated with a commercial benchtop Wasatch 785 Raman spectrometer configured with a free-space 50 μm input slit and a detector active between 200 and 2000 cm^−1^, corresponding to the fingerprint regime of tissue. The linear return fibre array was imaged onto the slit with a demagnification of 2 to fill the high f/1.3 aperture of the spectrometer fully. Excitation light was provided by a fibre-coupled, volume-holographic-grating (VHG) stabilised 785 nm laser diode (Thorlabs LP785-SAV50, Thorlabs, Inc., Newton, NY, USA) with a single-mode output, up to 50 mW of power, and a spectral linewidth of 0.25 nm. An 800 nm long-pass dielectric filter (Thorlabs FELH800, Thorlabs, Inc.) was positioned in the return path to prevent laser light from entering the spectrometer—thereby reducing stray light in the spectrometer and subsequently the noise floor of the Raman signal. For the experiments described here, 1 m long delivery and collection fibres were used. Shorter fibres would result in a lower FB; however, this length is practical in a laboratory setting and is also compatible with standard endoscopes used for upper endoscopy. 

**Figure 8:**
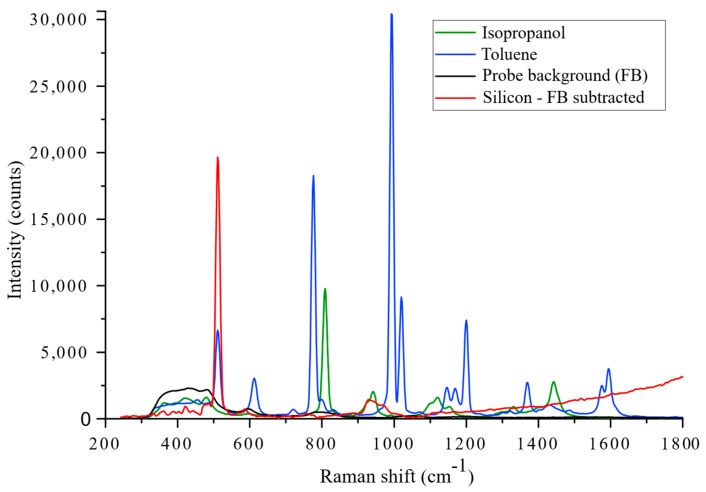
The Raman spectra of common Raman active reference materials obtained with the Raman probe. The spectra shown were measured over 5 s with 25 mW of laser power delivered onto the samples. The spectra are shown without any data processing applied with the exception of the silicon sample (red) which has had the fibre background (FB) (black) removed for clarity.

For the purpose of device development, relatively simple molecular materials such as hydrocarbons make ideal test samples because their associated Raman spectra are often well defined and catalogued. Additionally, the use of accessible materials allows for ease of comparison with other groups in the field. Figure 8 shows the measured Raman spectra of some common chemical species, namely toluene, isopropanol, and solid silicon, as well as the FB of the Raman probe itself. The spectra were acquired over 5 s with 25 mW of pump power measured at the distal end. When measuring the spectra of toluene and isopropanol, the probe was fully submerged. For silicon, the probe was suspended slightly above the material sample surface, and the FB was measured with the probe suspended in free space. Since the probe was designed to operate in contact with tissue, the sampling confinement and collection efficiency were expected to be slightly reduced in these cases due to spherical aberration at the probe tip. The results are presented without any background subtraction or data processing applied, with the exception of the silicon spectrum, which has had a normalised FB spectrum subtracted. The FB was more prominent in the silicon spectrum because the material was reflective and, so, more laser light was returned into the collection fibres. However, crystalline silicon has a very simple Raman spectrum with only a single dominant peak at around 520 cm^−1^ which allows the silica FB to be removed easily by first normalising the FB to a known silica peak in the measured silicon spectrum. For the submerged samples, the FB was low because the liquid partially index matched the tip of the probe and little light was back reflected into the return fibres.

**Figure 9:**
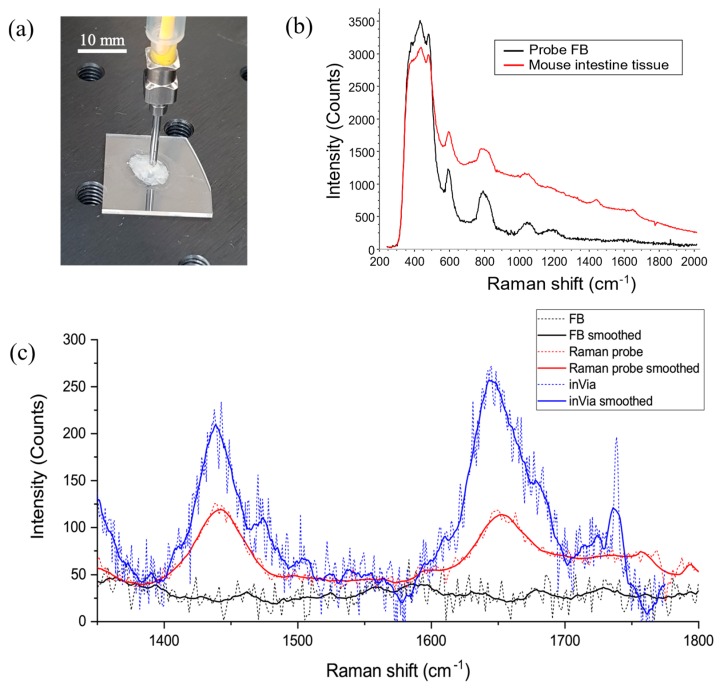
(**a**) A mouse intestine sample prepared on a fused silica substrate during spectral analysis with the Raman probe, embedded within the bore of a blunt needle, in light contact. (**b**) The average Raman spectra of eight tissue samples (red) and the FB (black) measured with the probe during 5 s acquisitions. (**c**) A portion of the spectrum between 1300 and 1800 cm^−1^ highlighting relevant tissue Raman peaks measured with the Raman probe (red) and compared with the same measurement performed with a commercial inVia Raman microscope (blue).

Further to the acquisition of simple chemical species, proof-of-principle Raman analysis was performed on ex vivo cancerous mouse intestine, which better represents the optical and molecular conditions of the intended sensing environment. The Apc(Min/+) mouse used is a well-established animal model for human colon cancer [44,45]. The tissue samples were prepared flat on fused silica substrates (which emit a minimal background fluorescence), as shown in Figure 9a. During spectral acquisition, the Raman probe was brought into gentle contact with the tissue surface and, as previously, 25 mW of laser power was delivered onto the tissue for an acquisition time of 5 s. Under these irradiation conditions, no tissue damage was observed throughout the experiments. Raman spectra were measured from several cancerous lesions as well as from visibly normal tissue; however, at this stage the aim was to identify diagnostically relevant tissue Raman peaks and not differentiate between benign and malignant tissue, which is typically only feasible on large datasets using robust statistical analysis. Figure 9b shows the averaged Raman spectrum of tissue (red) collected from 8 tissue sites without any data processing applied. The FB, shown in black, dominated the spectral profile with an additional broadband tissue-fluorescence contribution also apparent. Nonetheless, at least two significant Raman peaks were clearly distinguishable in the spectra, at approximately 1445 and 1660 cm^−1^. These peaks correspond primarily to CH_2_ bending (1400–1470 cm^−1^) and the amide I C=O stretching band (1645–1665 cm^−1^) [46], which are prominent molecular bonds found in organic matter. Studies have suggested that a ratiometric analysis of these Raman peaks might be used to differentiate malignant tissue from normal tissue [47].

To qualify the performance of the Raman probe further, spectral measurements were performed on the same samples with a commercial Renishaw inVia Reflex Raman microscope for comparison. A quantitative comparison of the systems is not robust as each spectrometer sensor behaves very differently; however, it does give an indication of the fibre probe’s potential beyond research. For comparative measurements, a 20×, 0.4 NA objective lens was used alongside a 785 nm laser source. Note that the optical power delivered onto the sample when using the Raman microscope was 38.7 mW, compared with 25.0 mW used with the Raman probe. The acquisition time was set to 5 s as with previous measurements. The average of eight spectra is shown in Figure 9c in blue alongside the spectra measured with the Raman probe in red, over the spectral range containing the two tissue peaks. For this plot, the spectra had moving-average baselines subtracted to reduce the contribution from fluorescence, and the spectra are displayed with and without third-order Savitzky–Golay smoothing (solid and dashed lines, respectively). 

The signal measured with the Raman probe was spectrally similar to that obtained with the inVia microscope, although noticeably less intense. The probe was designed to collect a signal from beneath the surface of the tissue, whereas the microscope was focused on the tissue surface, and so we might expect some reduction of the returned signal due to scattering within the tissue. Nonetheless, the results are promising, and we expect the signal strength to be sufficient to differentiate tissue in a clinical setting with some further optimisation; specifically, with the coincidence of the excitation and collection foci. 

The FB in the measured tissue Raman spectra was more severe than that for the liquid samples because tissue is more scattering and the underlaying silica substrate was partially reflective and therefore more excitation light was returned into the collection fibres. There are several techniques described in literature that have been used to reduce the FB and many of these are compatible with the Raman probe described here. Distal-end filtering may be used to “clean” the delivered pump light, block laser light from returning up the collection fibres, or both. Suitable filtering methods include incorporating miniaturised dielectric stacks into the probe housing [14,35,48], Bragg gratings [32,49,50] within the delivery fibre or probe bulk, hard-coating the optical fibre tips [31], background-free hollow-core fibres, or time gating the collected signal to remove the background from the tissue signal temporally [51]. 

The manufacturing method described here is particularly suitable for fabricating the DOS components as it is highly repeatable, automated, and offers integrated passive alignment capabilities. In order for the manufacture to be scaled up however, the fabrication time must be reduced from the few few hours currently to tens of minutes per part. Fortunately, there are several advanced techniques that may be employed to reduce the inscription time. These include using shaped wavefronts such as Bessel beams to modify large surface areas in fewer laser passes [52,53], multispot arrays to write multiple components simultaneously [21,54], and high-speed laser writing with galvo scanning mirrors [55]. Improving the manufacturability remains an active area of interest that we aim to investigate in future research. 

## 6. Conclusions

We have developed a miniaturised confocal Raman probe with a novel distal-end optical system and demonstrated that ultrafast laser-assisted etching is the ideal manufacturing tool for such a system, with potential for large-scale manufacture. The Raman probe had a collection efficiency of 71.3% ± 1.5% over a large 0.8 NA and a confocal sampling region 300 μm beyond the probe tip, coinciding with the depth at which precancers are expected to form in the epidermis. The probe has a small 960 μm form factor, which allows it to be delivered within a standard 17-gauge hypodermic needle or flexible endoscopic instrument channel, and is therefore suitable for deployment in several organs in the body. Passive optical fibre alignment to within ±0.83 μm was achieved using alignment slots laser written directly into the lens components. Microlenses with an optical quality surface finish were obtained, aided by a flame polishing technique to reduce etching roughness to ~2 nm, thereby minimising the noise contribution from scattering. 

Spectroscopic analysis was performed on common reference materials and highly distinguishable spectra were obtained after 5 s acquisitions with 25 mW of pump power at 785 nm. Proof-of-concept tissue spectral analysis of colorectal mouse tissue was also performed, and Raman peaks associated with CH_2_ bending and stretching modes and the amide I band were identified. With further optimisation to reduce acquisition times and the implementation of distal-end filtering, we expect the probe to be well-suited to an in vivo environment to aid clinical decision making at point-of-care.

## 7. Patents

The authors have protected aspects of this work through the following patent applications: CN110235036A; EP3574355A1; WO2018138490A1; US2019361174A1.
